# The Unfit

**Published:** 1911-10

**Authors:** 


### The Unfit.

In the April *Herald* we asked: "What Would the Isms Do if the Comma Bacillus Came Back?" The statistics recited showed that 23,800 Americans died from 1832 to 1853 (1500 deaths yearly average for the period) from the disease besides one-tenth of Cuba's population in one epidemic; that France lost 234,000, and England 16,000 during the same period; that Russia lost 800,000 and Hamburg 8000 in the single epidemic of 1892. These figures are to remind us what this little bacillus of Koch would do to us when given the keys to the nation's ports and allowed the free-handed and unimpeded culture of the empty-head doctrines and arguments of the isms opposing an official Department of Public Health.

At this writing the comma bacillus is not only in the port, but in the city of New York. Nine deaths to July 22d is the record with twelve cases of cholera still in the hospital. July 23d one case was discovered in Boston with twenty-five families and fifty children exposed. So far as known none of the noisy members of the League for Medical Freedom have rushed in with their slush fund or in person to turn a subduing hose on the conflagration. Mrs. Eddy's book has not been read to prove this little devil of a bacillus a myth and that the malingering sufferers are only in error. No homeo pellets have been cast on the waters, and the Eclectic Staff of Peace has not yet stroked the troubled waves. No daily headlines, at so much per, have beckoned the way out; but the plain, plodding every-day physician has turned his laboratory guns on these Asiatic bugs just as if they were real entities; just as if they could take heroic dosages; just as if they enjoyed a bi-vegetable and metallic medicant, and just as if they were in peaceful repose under the bulwark of the League for Medical Freedom as a preordination to the cause of the people. How futile would be the battle without government authority and generalship, and how great a disaster would again be recorded in a row of figures representing preventable deaths.

The great future of medicine is in the prevention of disease and its workings for the greatest good to all the people will be best conserved in the hands of the government that has made the United States a good place to live in. The mastery over yellow fever in Cuba and the digging of the Panama Canal made possible by medical brains shows the nation's need of progressive medicine. A national guidance of the great developing medical problems means fewer physicians and less medicine, but a more healthful people by keeping them well by prophylactic measures.

Perhaps no law ever became a law without argumentative opposition. Possibly some good, but much bad and indifferent resentment is opposing a Department of Public Health, but what seems to be the most pathetic exhibition of morbid psychology is illustrated in a self-sacrificing clinical address to the United States Senate July 6th. The speaker, a Senator from a proud State, detailed his life of invalidism, near unto death, beyond the best medical aid, and all because of a displaced vertebra pressing on the spinal cord. He detailed fifteen years of helpless invalidism in the wife, dating back twenty-five years. He related descriptively the pangs of "Love's Labor Lost" (almost) in a drunken, debauched son and a final glorious restoration through Eddyism. The *Los Angeles Daily Times* says Senator Work "delivers most peculiar speech ever heard in Congress." Is it peculiar that a peculiar Senator should be peculiarly discovered by peculiarly interested home people when delivering himself of a peculiar speech on a peculiar subject to a peculiar Congress that would listen to such tommyrot? Is this exploitation a different manifestation of a basic psychologic status that, in other days, made our nation mourn? Will the propagation of its kind make the well mourn the dead after pestilence and mourn those who are not dead because of being a pestilence in their living?

This would seem a charitable view, for we are too obtuse for a visionary spinal cord pressure to appeal to us. We could fancy a slight, continuous or urgent pressure between the grandiose center of finance and a cohesive, but restless senatorial gluteus maximus actively stimulating opportune moments. Again we can surmise a sublaxation between the crista galli and the foramen mangum producing psychologic phantoms and alcoholic degenerates, but the spinal dislocation pressure founders our receptive. Candidly, setting aside charity, is the whole thing an accouchement monstrosity? That is has some mysterious person at some time, in some bodies lonely hay manger been delivered, on a starless night, of a plain lie?

It is hoped, if the *Los Angeles Daily Times* of July 7th is correct, that the proud California mountains will be deluged with rivulets clear long before they are strained again in the birth of another Senatorial Unfit for the making of laws to govern the health of the nation.—Burnett, in *Medical Herald* for August.

